# Lowering hippocampal miR-29a expression slows cognitive decline and reduces beta-amyloid deposition in 5xFAD mice

**DOI:** 10.21203/rs.3.rs-3235257/v1

**Published:** 2023-08-16

**Authors:** Zhen Mei, Jiaqi Liu, Jason P Schroeder, David Weinshenker, Duc M. Duong, Nicholas T. Seyfried, Yujing Li, Peng Jin, Aliza P. Wingo, Thomas S. Wingo

**Affiliations:** Emory University

**Keywords:** miR-29a, Cognition, Beta-amyloid, Neuroinflammation, Wdfy1

## Abstract

microRNA-29a (miR-29a) increases with age in humans and mice, and, in the brain, it has a role in neuronal maturation and response to inflammation. We previously associated higher miR-29a levels in human brain with faster antemortem cognitive decline, suggesting that lowering miR-29a levels could ameliorate memory impairment in the 5xFAD AD mouse model. To test this hypothesis, we generated an adeno-associated virus (AAV) expressing GFP and a miR-29a “sponge” or empty vector. We found that the AAV expressing miR-29a sponge functionally reduced miR-29a levels, and improved measures of memory in the Morris water maze and fear condition paradigms when sponge delivered to hippocampi of 5XFAD and WT mice. miR-29a sponge expression significantly reduced hippocampal beta-amyloid deposition in 5XFAD mice and lowered astrocyte and microglia activation in both 5XFAD and WT mice. Using transcriptomic and proteomic sequencing, we identified Plxna1 and Wdfy1 as putative effectors at the transcript and protein level in WT and 5XFAD mice, respectively. These data indicate that miR-29a promotes AD-like neuropathology and negatively regulates cognition, making it and its target genes attractive therapeutic targets for the treatment of neurodegenerative disease.

## Introduction

Alzheimer’s disease (AD) is the most common cause of dementia. Recent epidemiological data indicate that the number of people with AD worldwide will increase to 131.5 million by 2050^1^. AD is a neurodegenerative disorder characterized by progressive cognitive decline, neuronal loss, and brain pathology. Its neuropathological hallmarks are neuritic plaques comprised of amyloid-β aggregates and tau-containing neurofibrillary tangles. The pathologic hallmarks of AD typically coexist with other age-related pathologies (e.g., Lewy bodies and vascular disease), which are common in people over 70 years and collectively account for only about 40% of the cognitive decline^2,3^. This observation has led to investigations of other potential contributing factors, including the microRNAs (miRNAs)^4 5^.

miRNAs are small non-coding RNAs that regulate gene expression by either mRNA degradation or translation inhibition^6^. Each miRNA can target hundreds of transcripts, thereby potentially exerting a widespread influence on the transcriptional landscape of a cell. Certain miRNAs are implicated in synaptic plasticity, neuronal survival, and aggregation of neurodegenerative pathologies^7–9^. Our previous work identified miRNAs associated with cognitive trajectory using postmortem brains specimens in a global miRNA association study^5^. Among these, miR-29a was the second most significantly associated miRNA with cognitive trajectory, even after accounting for the eight measured age-related brain pathologies^5^. Because higher miR-29a level was associated with faster cognitive decline^5^, we hypothesized that lowering miR-29a levels would improve cognitive performance.

Here we aimed to test our hypothesis that reducing miR-29a would slow cognitive decline using 5XFAD transgenic mice and their wild-type (WT) littermates, and to investigate the downstream effects of lowering miR-29a at the transcript and protein level. We first designed a miR-29a sponge to “soak up” the microribonucleoprotein complexes (microRNPs) loaded with miR-29a and thereby derepress its downstream targets. AAV expressing miR-29a sponge or control AAV were stereotaxically injected into hippocampi of 5XFAD and WT mice. Cognition was assessed by the Morris water maze and fear conditioning, amyloid deposition and activated astrocytes and microglia were evaluated by immunofluorescence staining, and RNA-sequencing and deep proteomic sequencing were performed to identify the downstream effectors of miR-29a. We found that miR-29a loss-of-function improved learning and memory and attenuated amyloid deposition and immune cell activation in 5XFAD and WT mice. Transcriptomic and proteomic analyses identified targets of miR-29a and suggest a role for miR-29a in modulating neuroinflammation.

## Methods

### Mice

Female 5XFAD and C57BL/6 mice used in the studies were group housed (maximum of 5 animals per cage) in the Department of Animal Resources at Emory University under standard conditions. 5XFAD transgenic mice (The Jackson Laboratory, #034848) were purchased and maintained as hemizygotes on a C57BL/6 background. 5XFAD transgenic mice were confirmed by polymerase chain reaction and non-transgenic WT littermates were used as controls. All experiments were conducted in strict accordance with the National Institutes of Health Guide for the Care and Use of Laboratory Animals and approved by the Emory Institutional Animal Care and Use Committee.

### Construction of miR-29a mimic

miR-29a mimic was constructed by first amplifying fragment encoding pre-miR-29a from the genomic DNA isolated from HEK-293T cells. The PCR product was then purified, digested with EcoRI and XbaI, and inserted into digested pAAV-MCS vector (Addgene, VPK-410). The construct sequenced was verified by Sanger sequencing. The primer sequences for miR-29a mimic are given in Supplementary Table 1.

### miR-29a sponge design and cloning

miR-29a sponge was seven tandemly arrayed miR-29a binding sites separated by a 4-nt spacer, each of which was perfectly complementary in the seed region but with a bulge at positions 9–12 to prevent degradation by Argonaute 2^10^. We annealed, ligated, gel purified and cloned miR-29a sponge into 3’UTR of psiCHECK-2 vector (Promega, C8021) and pAAV-GFP vector (Cell biolabs, AAV-400), respectively. All constructs were verified by Sanger sequencing. The primer sequences for miR-29a are given in Supplementary Table 1.

### Luciferase assays

We plated HEK-293T cells into 24-well plates the day before transfection and transfected them in triplicate with psiCHECK-2 vector containing miR-29a sponge together with miR-29a mimic or empty vector (pAAV-MCS) at a ratio of 1:20 (300ng total DNA/well). Lipofectamine 2000 (Invitrogen, 11668019) was used as the transfection reagent. Cells were lysed in passive lysis buffer 48 h after transfection and assayed in triplicate using the Dual-Luciferase Reporter Assay System (Promega, E1910). Renilla luciferase activity was normalized to Firefly luciferase activity measured on a GloMax 96 microplate luminometer (Promega, E6521) and was then calculated relative to the negative control in each independent replicate.

### Western blots

HEK-293T cells were plated into 6-well plate the day before transfection. miR-29a mimic together with pAAV-GFP vector containing miR-29a Sponge or empty vector (pAAV-GFP) were co-transfected into HEK-293T cells at a ratio of 20:1 with lipofectamine 2000 (1000ng total DNA/well). Fluorescence microscopy was used to check the percentage of GFP positive cells 24 h after transfection. 48 h after transfection, cells were lysed in RIPA buffer (Thermo Fisher Scientific, 89900) supplemented with Protease Inhibitor Cocktail (Roche, 4693159001). Protein concentrations were determined with the BCA Protein Assay kit (Thermo Scientific, 23235) according to the manufacturer’s instruction. Equal amounts of total proteins were resolved on a 10% Mini-Protean TGX precast gel (Bio-Rad, 4561033), and transferred to a PVDF membrane (Bio-Rad,1704156) with the Trans-Blot Turbo transfer system (Bio-Rad). The membranes were incubated with blocking solution (5% skim milk in Phosphate-buffered saline with 1% Tween-20) for 1 h, blotted by a blocking solution containing a primary antibody (DNMT3A, Cell Signaling Technology, #3598, 1:1000; GAPDH, Invitrogen, #39-8600, 1:10000) overnight at 4°C, and incubated with a horseradish peroxidase conjugated secondary antibody for 1 h. Proteins were visualized by ECL prime detection reagent (Cytiva, RPN2232) and ChemiDoc imaging system (Bio-Rad). Immunoreactive bands were quantified with ImageJ software. The experiment was performed in triplicate.

### Adeno-associated virus (AAV) production and stereotaxic injection

The plasmid templates for AAV generation were pAAV-GFP vector containing miR-29a sponge or the empty vector. AAV-miR-29a sponge and control AAV were generated by Emory University Viral Core. Before AAV injection, female 5XFAD and WT mice were anesthetized and placed in a stereotaxic frame. After a skin incision was made, holes were drilled at x (± 1.5 mm from bregma) and y (− 2.0 mm from bregma). AAV-miR-29a sponge or control AAV were injected into the left and right hippocampi (z = − 1.9 mm from bregma) respectively, with 6.2X10^10^ total viral particles per side and delivered at a rate of 0.2μL/min. The syringe was left in place for 5 min and withdrawn slowly after each injection. When the injection was complete, skin was sutured and sterilized.

### Morris water maze

Experiments were conducted by the Emory University Rodent Behavioral Core by trained personnel who were blinded to the mouse condition. In a circular 52-inch-diameter tank filled with opaque water kept at 23°C with a hidden circular platform (30 cm diameter) present 1 cm below the water in the northwestern quadrant of the tank, each mouse had four training trials to find the platform per day over 5 consecutive days. Each training trial lasted a maximum of 60 s. If a mouse did not find the platform in time, it was manually guided to it and placed on the platform for 10 s. Escape latency to the platform as well as swim speed were recorded by an automated tracking system (TopScan, CleverSys). A probe trial was conducted on the sixth day where the platform was removed, and the mice were released from the south start point and allowed to swim for 60 s. The tracking system recorded the percentage of search time in the quadrant where the platform was previously located.

### Fear conditioning

Fear conditioning was conducted by the Emory University Rodent Behavioral Core by trained personnel who were blinded to the mouse condition. Fear conditioning occurred over 3 consecutive days in a chamber (H10-11M-TC, Coulbourn Instruments) equipped with a house light, a speaker, a ceiling-mounted camera and an electric grid shock floor that could be replaced with a non-shock wire mesh floor. Fear conditioning training on day 1 began with a 3 min acclimatization period followed by 3 tone-shock pairings during which the tone lasted 20 s and was co-terminated with a 3s, 0.5mA foot shock. Mouse behavior was recorded for 60 s after a tone-shock pairing before the next round. Contextual fear testing on day 2 was conducted in the same chamber as day 1 without any tone or shock. Cued fear testing on day 3 was conducted in a different chamber with a non-shock wire mesh floor and began with a 3 min acclimatization period followed by a 5 min tone without any shock. FreezeFrame software (Coulbourn Instruments) was used to record freezing behavior and the percentage of freezing time was determined.

### RNA isolation and real-time quantitative PCR

Samples were homogenized in TRIzol reagent (Thermo Fisher Scientific, 15596018) and shaken for 15 s after addition of chloroform. The samples were transferred to pre-spun Phase Lock Gel-Heavy tubes (Quanta bio, 2302830) and incubated at RT for 5 min and then centrifuged at 12,000 g/4°C for 15 min. The upper phase aqueous solution was collected in a fresh tube and RNA was precipitated by isopropanol. Samples were gently mixed and left at −80°C overnight and then centrifuged at 14,000 rpm/4°C for 25 min. RNA pellet was washed twice in 75% ethanol and resuspended in nuclease-free water. miR-29a was reverse transcribed to cDNA using miR-29a specific primers from TaqMan (Thermo Fisher Scientific, Assay ID: 002112). Real-time polymerase chain reaction was performed using the Applied Biosystems TaqMan Gene Expression assay following the manufacturer’s instruction. Data were analyzed by the ΔΔCt methods using U6 as an endogenous control.

### Tissue preparation

After cervical dislocation, mouse brains were removed and dissected at the midline. For biochemical analysis, hippocampi were dissociated and immediately snap-frozen in liquid nitrogen and stored at −80°C for protein and RNA sequencing. For immunofluorescence staining, mice were anesthetized and transcardially perfused with 0.9% sodium chloride and then fixed in ice-cold 4% paraformaldehyde in 1× PBS. The brains were removed and postfixed in 4% paraformaldehyde overnight at 4°C and transferred to 30% sucrose at 4°C for 48 h before being embedded for cryostat sectioning.

### Immunofluorescence staining

Mouse brains were embedded with optimal cutting temperature compound (Tissue-Tek, 4583) and cut into serial 10-μm-thick coronal sections with a cryostat (Leica Biosystems). The sections were washed three times in 1XPBS for 5 min each and incubated with blocking buffer (PBS with 10% normal goat serum and 0.25% Triton X-100) for 1 h at RT. The sections were then incubated with primary antibodies overnight at 4°C, followed by incubation with Alexa-fluorophore-conjugated secondary antibodies (Thermo Fisher Scientific, A-11004, A-11011, 1:500) for 1 h at RT in the dark. Sections were rinsed and mounted onto slides using Vectashield mounting medium with DAPI (Vector Laboratories, H-1200). The following primary antibodies were used for immunofluorescence staining: anti-beta amyloid (Abcam, ab2539, 1:200); anti-Iba1 (Wako Chemicals, 019-19741, 1:200); and anti-GFAP (Abcam, ab7260, 1:200). Immunoreactivity (IR) was calculated as mean gray value (area of IR within ROI divided by total area of ROI) within ImageJ as previously described^11^. Images were obtained on a ZEISS 710 confocal laser-scanning microscope.

### RNA seq data processing

Total RNA used in the sequencing study was isolated from the hippocampus using TRIzol reagent (Thermo Fisher Scientific, 15596018). RNA quality was measured on an Agilent 2100 Bioanalyzer system based on the 28S/18S ratio and the RNA integrity number (RIN). For each sample, 1 μg RNA was used to construct sequencing libraries using Illumina’s TruSeq RNA Sample Prep Kit. Samples were sequenced on Illumina’s HiSeq 2000 system with a sequencing depth of 40M total reads per sample (20M each direction), producing sequencing result in FastQ format. The QC on the sequence reads was done with FastQC and all samples were carried forward in the analysis. Sequenced data were aligned to mouse reference genome using STAR aligner version 2.7.3a^12^ and STAR produces a read count file for each sample using the algorithm of htseq-count^13^ with default settings.

### Gene differential expression analysis

The differential expression analysis across experiment and control groups was implemented in R (version 4.1.2). Variance stabilizing transformation (VST) function offered by DESeq2 R package (version 1.32.0)^14^ was used to log_2_ transform the raw counts, normalize for library sizes, and reduce heteroskedasticity. The Surrogate Variable Analysis (SVA) method in the sva R package (version 3.40.0)^15^ was used to detect potentially hidden variables from the normalized data. Given our sample size is small (n = 8), we only included the first surrogate variable (SV1) in the design matrix^15^. The R package DESeq2 was used to perform the differential expression analysis adjusted for SV1. The Benjamini-Hochberg method was used to control for the false discovery rate (FDR) and considered significant at FDR less than 0.1. The R package clusterProfiler^16^ (version 4.0.5) was used to perform gene ontology enrichment analysis for the significant genes following instructions in the package vignette. GO terms were considered significant at an FDR adjusted p-value less than 0.05.

### Protein digestion and TMT labeling

Each mouse hippocampus sample was digested individually using the EasyPep^™^ mini sample preparation kit according to manufacturer instructions (ThermoFisher Scientific, A40006). Briefly, each sample was homogenized in 200 uL kit lysis buffer with Halt protease inhibitors and nuclease. A protein concentration assay was performed and 80ug went through digestion and desalting. Resulting peptides were desalted with a Sep-Pak C18 column (Waters, WAT054945) and dried under vacuum. Peptides were reconstituted in 100ul of 100mM triethyl ammonium bicarbonate (TEAB) and labeling performed as previously described^17,18^. One batch of 16-plex TMTPro isobaric tags (Thermofisher Scientific, A44520) was used to label all 16 samples. All 16 channels were then combined and dried by SpeedVac (LabConco) to approximately 100 μL and diluted with 1 mL of 0.1% (vol/vol) TFA, then acidified to a final concentration of 1% (vol/vol) FA and 0.1% (vol/vol) TFA. Peptides were desalted with a 60 mg HLB plate (Waters). The eluates were then dried to completeness.

### High pH Fractionation

High pH fractionation was performed essentially as described^19^ with slight modification. Dried samples were re-suspended in high pH loading buffer (0.07% vol/vol NH4OH, 0.045% vol/vol FA, 2% vol/vol ACN) and loaded onto a Water’s BEH (2.1mm × 150 mm with 1.7 μm beads). An Thermo Vanquish UPLC system was used to carry out the fractionation. Solvent A consisted of 0.0175% (vol/vol) NH4OH, 0.01125% (vol/vol) FA, and 2% (vol/vol) ACN; solvent B consisted of 0.0175% (vol/vol) NH4OH, 0.01125% (vol/vol) FA, and 90% (vol/vol) ACN. The sample elution was performed over a 25 min gradient with a flow rate of 0.6 mL/min with a gradient from 0 to 50% B. A total of 96 individual equal volume fractions were collected across the gradient and dried to completeness using a vacuum centrifugation.

### Liquid Chromatography Tandem Mass Spectrometry

All samples (~ 1ug for each fraction) were loaded and eluted using Dionex Ultimate 3000 RSLCnano (Thermofisher Scientific) an in-house packed 15 cm, 100 μm i.d. capillary column with 1.9 μm Reprosil-Pur C18 beads (Dr. Maisch, Ammerbuch, Germany) using a 23 min gradient. Mass spectrometry was performed with a high-field asymmetric waveform ion mobility spectrometry (FAIMS) Pro equipped Orbitrap Eclipse (Thermo) in positive ion mode using data-dependent acquisition with 2 second top speed cycles. Each cycle consisted of one full MS scan followed by as many MS/MS events that could fit within the given 2 second cycle time limit. MS scans were collected at a resolution of 120,000 (410–1600 m/z range, 4×10^5 AGC, 50 ms maximum ion injection time, FAIMS compensation voltage of −45). All higher energy collision-induced dissociation (HCD) MS/MS spectra were acquired at a resolution of 30,000 (0.7 m/z isolation width, 35% collision energy, 1.25×10^5 AGC target, 54 ms maximum ion time, TurboTMT on). Dynamic exclusion was set to exclude previously sequenced peaks for 20 seconds within a 10-ppm isolation window.

### Protein identification and quantification

All raw files were searched using Thermo’s Proteome Discoverer suite (version 2.4.1.15) with Sequest HT. The spectra were searched against a mouse uniprot database downloaded August 2020 (91414 target sequences). Search parameters included 20ppm precursor mass window, 0.05 Da product mass window, dynamic modifications methione (+ 15.995 Da), deamidated asparagine and glutamine (+ 0.984 Da), phosphorylated serine, threonine and tyrosine (+ 79.966 Da), and static modifications for carbamidomethyl cysteines (+ 57.021 Da) and N-terminal and Lysine-tagged TMT (+ 304.207 Da). Percolator was used filter PSMs to 0.1% FDR. Peptides were group using strict parsimony and only razor and unique peptides were used for protein level quantitation. Reporter ions were quantified from MS2 scans using an integration tolerance of 20 ppm with the most confident centroid setting. Only unique and razor (i.e., parsimonious) peptides were considered for quantification.

### Protein differential expression analysis

The normalization and differential analysis of the proteomics data were performed in R (version 4.1.2). We included proteins with TMT abundance values in at least 50% replicates per group and display a high protein FDR confidence (FDR < 0.01). To normalize the raw data, for each sample, each protein’s abundance was first divided by the sum of abundance values of all the proteins profiled for that sample, followed by the log2 transformation. For each protein, we then constructed a linear model of normalized abundance as a function of group. The p values were adjusted for multiple comparisons using the FDR method. We next selected proteins that are predicted targets of miR-29a based on miRDB^20,21^ database and then performed the differential expression analysis. Proteins were declared to be significant at an FDR adjusted p-value less than 0.1 using the Benjamini-Hochberg control for the FDR.

### Experimental design and statistical analysis

For AAV injection, female mice at 6–7 months of age (WT or 5×FAD) were injected with control AAV or AAV-miR-29a sponge. For simplicity, we refer the injection groups as WT-Control, WT-Sponge, FAD-Control and FAD-Sponge. The number of mice in each group was: WT-Control (n = 12), WT-Sponge (n = 11), FAD-Control (n = 9), FAD-Sponge (n = 10). The behavioral tests were performed 3 months after injection. We then randomly selected 3 mice from each group for immunofluorescence staining. For RNA-Seq and proteomics analyses, 4 mice were randomly selected from each group and one side of hippocampus was harvested for RNA-Seq analysis and the other side was for proteomics.

The expression of miR-29a and behavioral testing (i.e., Morris water maze and fear conditioning) were tested using two-way ANOVA or repeated measures (RM) ANOVA, followed by *post hoc* methods to control for multiple comparisons. Values were considered significant at *p* < 0.05 and a tendency at *p* < 0.1. Calculations were performed and figures created using Prism version 8.3 for Windows.

## Results

### Endogenous expression level of miR-29a in mouse hippocampus

**Figure 1:**
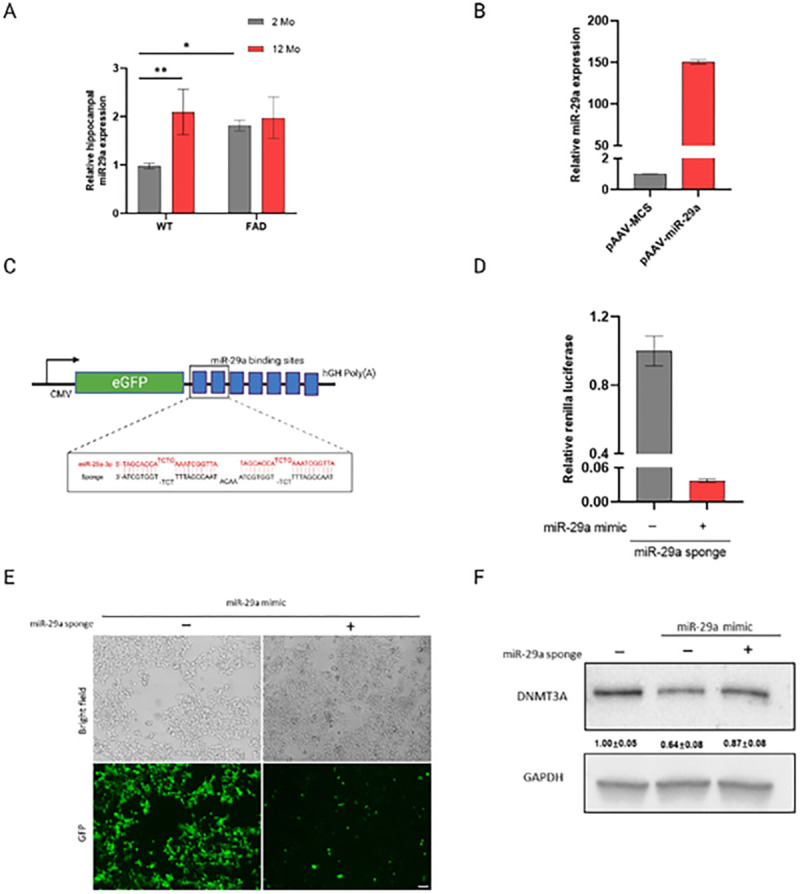
miR-29a expression in mouse hippocampus and miR-29a sponge validation in vitro. **A)** miR-29a was determined in the hippocampus of 2 and12-month-old 5XFAD and WT mice by real-time RT-PCR. U6 snRNA was used as the endogenous control. Data was analyzed via Two-way ANOVA (age by genotype) with Sidak’s *post hoc* tests. Bars are mean ± standard deviation (SD) with n=3 per group; *p<0.05, **p<0.01. **B)** Relative expression level of mature miR-29a in HEK-293T cells transfected with miR-29a mimic (pAAV-miR-29a) and control vector (pAAV-MCS).**C)** Schematic representation of miR-29a sponge mechanism and design. Sponge sequence was shown as black color and target miRNA was shown as red. **D)**Renilla luciferase activity was assayed relative to firefly luciferase activity in 293T cells transfected with psiCHECK2 vector containing miR-29a sponge and miR-29a mimic (pAAV-miR-29a) or control vector (pAAV-MCS). **E)** Cell morphology and GFP expression upon transfection of miR-29a mimic (pAAV-miR-29a) with pAAV-GFP vector containing miR-29a sponge or empty vector (pAAV-GFP), as observed by light and fluorescent microscopy. Scale bars represent 100 μm. **F)** Western blot analysis of DNMT3A upon transfection of miR-29a mimic with or without miR-29a sponge. Experiments were performed 3 times and one representative western blot is shown. Quantification of western blots is provided under each blot. Data represents fold change relative to no treatment group ± SD. GAPDH was used as a loading control.

The endogenous expression of miR-29a was measured in the hippocampi of young (2-month-old) and aged (12-month-old) 5XFAD and WT mice, respectively (Fig. 1A). Two-way ANOVA revealed there was a main effect of age (*F*_(1, 8)_ = 11.66, *p* = 0.0092) and an age × genotype interaction (*F*_(1, 8)_ = 6.531, *p* = 0.0339). *Post hoc* Sidak’s tests showed 12-month-old WT mice had significantly higher level of miR-29a than 2-month-old WT (*t*_(8)_ = 4.221, *p* = 0.0058), while there was no significant difference between young and aged 5XFAD mice. Within genotype, *post hoc* Šidák correction showed 2-month-old 5XFAD displayed higher miR-29a than 2-month-old WT mice (*t*_(8)_ = 3.164, *p* = 0.0265), while there was no difference between 5XFAD and WT mice at 12-months. These data suggested miR-29a increased with age specifically in WT mice and miR-29a displayed a higher expression level in young 5XFAD mice.

### Testing molecular tools to investigate miR-29a

The overexpression of miR-29a by the miR-29a mimic was confirmed in HEK-293T cells (Fig. 1B). Yet, for this study, we focused on miRNA loss-of function to study the role of miR-29a because it reveals miRNA functions that depend on physiological levels, while miRNA overexpression can result in repression of non-physiological mRNA targets^22^. Thus, we constructed a miR-29a sponge with seven tandemly arrayed miR-29a binding sites to functionally down-regulate the level of miR-29a (Fig. 1C). To investigate the efficacy of the miR-29a sponge, we cloned miR-29a sponge into the 3’UTR of the Renilla luciferase gene of the psiCHECK2 vector and co-transfected the miR-29a sponge with or without miR-29a mimic in HEK-293T cells (Fig. 1D). As expected, we observed a decreased Renilla to Firefly ratio upon transfection with miR-29a sponge and miR-29a mimic (Fig. 1D), confirming the effectiveness of the sponge transcript binding to the miR-29a.

To test the ability of the miR-29a sponge to derepress downstream targets, we next assayed the protein Dnmt3a, a well-validated target of miR-29^23–25^. We cloned the miR-29a sponge into the 3’UTR of the GFP gene of pAAV-GFP vector. miR-29a mimic with or without the sponge construct were then co-transfected HEK-293T cells, and cells were observed for GFP expression at 24h using light and fluorescence microscopy. Figure 1E shows that GFP expression of the construct containing miR-29a sponge was visibly repressed upon transfection with miR-29a mimic relative to GFP control construct. We harvested the cells at 48h and assayed the protein Dnmt3a by Western blot. As shown in Fig. 1F, Dnmt3a expression decreased about 40% upon miR-29a overexpression and miR-29a sponge rescued the target by about 20%. These results further confirmed miR-29a sponge acted as a competitive regulator that sequestered miR-29a and preventing miRNA/mRNA interaction.

### Testing effect of miR-29a sponge in mouse hippocampi on learning and memory

**Figure 2:**
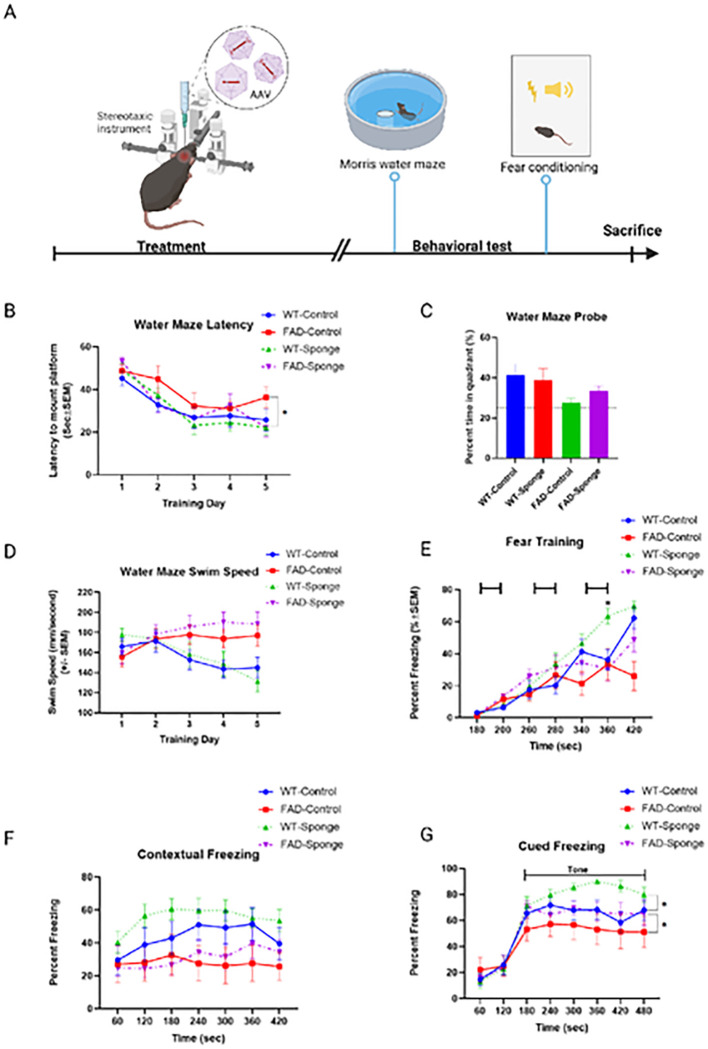
Results of Morris water maze and fear conditioning. **A)**Time line of experiments. **B)** Latency to find the platform. **C)** Water maze probe trial. **D)** Swim speed during 5 days of training trial. **E)**Fear training with 3 tone-shock pairings. **F-G)** Percentage time spent freezing following re-exposure to the shock-associated context or tone. Data are mean ± SEM with n=9–12 per group. Data were analyzed via two-way repeated measures ANOVA (time × group) or three-way repeated measures ANOVA (time × group × genotype). Water maze probe trial was analyzed via two-way ANOVA (genotype by group). *p<0.05.

To determine whether functionally downregulating the level of miR-29a impacted cognitive performance, we injected control AAV or AAV-miR-29a sponge into the hippocampi of 5XFAD and WT mice, respectively. The potential effect of derepressing miR-29a targets on learning and memory was tested using the Morris water maze and fear conditioning with the more stressful tasks performed first (Fig. 2A).

### miR-29a sponge ameliorated spatial learning in 5XFAD mice

The Morris water maze is considered a hippocampal-dependent learning and memory task. Mice were first trained for 5 consecutive days to learn the location of a hidden platform, and escape latency and swim speed were recorded. In the subsequent probe trial, mice were returned to the maze without the platform and the amount of time they spent in the platform quadrant was recorded.

All groups showed progressively shorter escape latencies over the 5 training days, and FAD-sponge generally showed lower latencies compared to FAD-control (Fig. 2B). Two-way repeated measures (RM) ANOVA between FAD-Sponge and FAD-Control revealed a main effect of time (*F*_(3.596, 68.33)_ = 12.4, *p* < 0.0001) and a time × group interaction (*F*_(4, 76)_ = 2.512, *p* = 0.0485). *Post hoc* Dunnett’s test showed that compared with day 1 of training, FAD-Sponge had significantly lower escape latency starting from day 2 (*t*_(10)_ = 7.136, *p* = 0.0001) and the remaining training days (day3, *t*_(10)_ = 5.112, *p* = 0.0016, day4, *t*_(10)_ = 4.598, *p* = 0.0033, and day 5, *t*_(10)_ = 6.864, *p* = 0.0002, respectively). By contrast, FAD-Control failed to demonstrate significant learning until day 4 of training (*t*_(9)_ = 3.519, *p* = 0.0208). In WT mice, there was a main effect of time (*F*_(2.468, 51.83)_ = 15.42, *p* < 0.0001), but no effect of time × group interaction. In the probe trial, all groups spent more than 25% of their time (i.e., greater than chance) swimming in the quadrant previously containing the platform, and WT mice spent significantly more time compared with 5XFAD mice (main effect of genotype: *F*_(1, 40)_ = 4.685, *p* = 0.0364). Although the FAD-Sponge mice tended to spend more time in the target quadrant than the FAD-Control mice, there were no significant effects of group or group × genotype interaction (Fig. 2C). We found no evidence for differences in swim speed between control or sponge conditions in either WT or FAD mice using a two-way ANOVA, but 5XFAD mice swam faster than WT mice (main effect of genotype: *F*_(1, 20)_ = 4.892, *p* = 0.0388, Fig. 2D). Taken together, these results indicate downregulating the miR-29a level can ameliorate spatial learning deficits in 5XFAD mice.

### miR-29a sponge improved cued fear memory in WT mice

We next assessed mice in fear conditioning, a hippocampal-dependent measure of associative learning and memory. During fear training, mice were placed in the fear conditioning chambers and exposed to 3 tone-shock pairings on day 1, and the percentage of freezing was recorded. The following day, mice were returned to the same chamber in the absence of tone or shock to assess contextual memory. On the third day of training, mice were placed in a novel chamber, and freezing in response to the tone alone was recorded to test cued fear memory.

During fear training, all groups showed learning in response to tone-shock pairings (Fig. 2E). RM two-way ANOVA between WT-Sponge and WT-Control revealed a main effect of time (*F*_(3.781, 79.41)_ = 68.39, *p* < 0.0001) and a significant time × group interaction (*F*_(6, 126)_ = 3.01, *p* = 0.0088). Post hoc Sidak’s test showed WT-Sponge froze significantly more at the 360 second timepoint compared with WT-Control (*t*_(20.25)_ = 3.414, *p* = 0.0189). FAD-Sponge and FAD-Control also showed more freezing with more training time (*F*_(2.938, 49.94)_ = 14.07, *p* < 0.0001), but there was no significant time × group interaction. For contextual memory, WT mice froze significantly more than 5XFAD mice (main effect of genotype: *F*_(1, 20)_ = 6.102, *p* = 0.0226), but neither WT- nor FAD-sponge showed significant differences compared with their control groups (Fig. 2F). All groups displayed cued freezing with the presentation of the tone on the third day of training (Fig. 2G). WT mice displayed more cued freezing than 5XFAD mice (main effect of genotype: *F*_(1, 20)_ = 4.462, *p* = 0.0474), and WT- and FAD-sponge groups showed significant differences compared with their control groups (main effect of group: *F*_(1, 20)_ = 4.668, *p* = 0.043). Overall, miR-29a sponge improved cued fear memory in both WT and 5XFAD mice.

### miR-29a sponge attenuated amyloid deposition and gliosis

**Figure 3:**
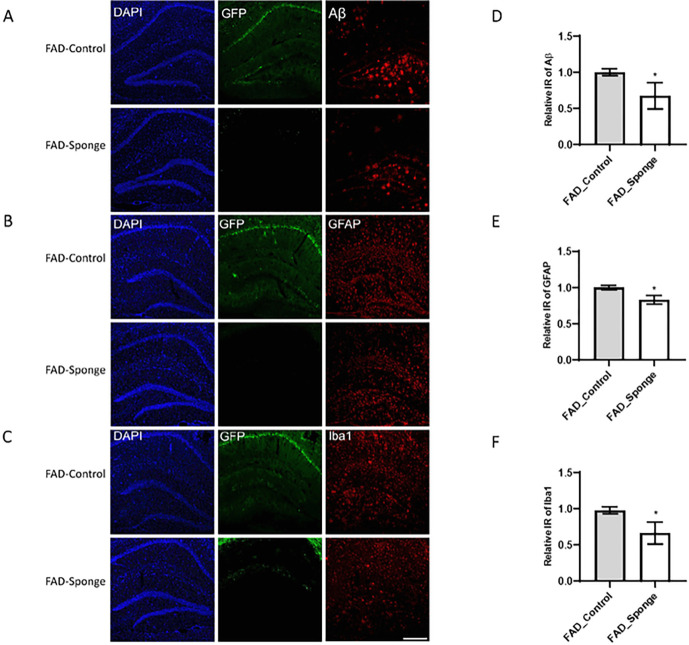
miR-29a sponge attenuates amyloid deposition and decreases activated astrocytes and microglia in the hippocampus of 5XFAD mice. **A-C)**Representative immunofluorescence images. **D-F)** Quantification of Aβ, GFAP and Iba1 immunoreactivity. Data were analyzed via t-test. Bars are mean ± SD. N=3 per group. Scale bars represent 100μm; *p<0.05.

**Figure 4:**
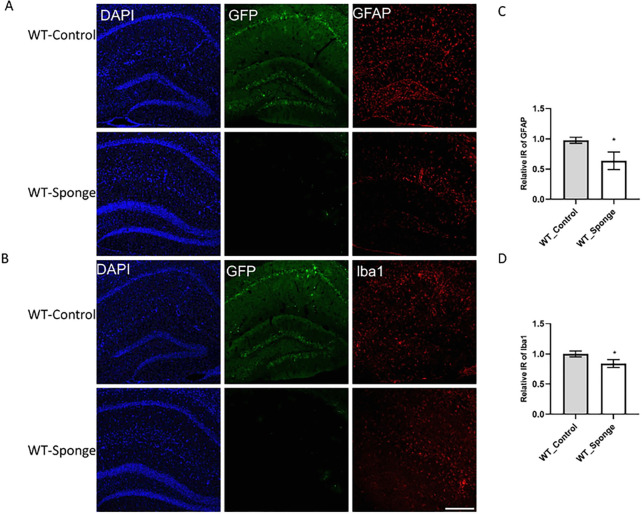
miR-29a sponge decreases activated astrocytes and microglia in the hippocampus of WT mice. **A-B)** Representative immunofluorescence images. **C-D)** Quantification of GFAP and Iba1 immunoreactivity. Data were analyzed via t-test. Bars are mean ± SD. N=3 per group. Scale bars represent 100 μm; *p<0.05.

To identify potential mechanisms for the amelioration of behavioral outcomes, we evaluated the hippocampi for beta-amyloid accumulation and neuroinflammation. Beta-amyloid deposition was evaluated exclusively in 5XFAD mice, since WT mice do not show any of this pathology. Robust amyloid plaque deposition was in the hippocampus of FAD-Control mice, which was significantly reduced in FAD-Sponge mice (Fig. 3A, t _(4)_ = 2.983, *p* = 0.0406). Neuroinflammation detected by the presence markers for activated astrocytes and microglia^26^. Both FAD- and WT-sponge mice showed decreased levels of GFAP immunoreactivity compared with their control groups (5XFAD, *t*_(4)_ = 4.365, *p* = 0.012, WT, *t*_(4)_ = 3.869, *p* = 0.018, Fig. 3B, Fig. 4A). For microglia, both FAD- and WT-sponge mice showed attenuated microglial activation (5XFAD, *t*_(4)_ = 3.394, *p* = 0.0274, WT, *t*_(4)_ = 3.361, *p* = 0.0283, Fig. 3C, Fig. 4B). GFP expression was visibly repressed in both FAD- and WT-Sponge groups relative to their control groups, further confirmed the efficacy of miR-29a sponge. These data demonstrate that miR-29a sponge attenuated amyloid burden and neuroinflammation in 5XFAD and WT mice.

### Downstream effectors of miR-29a

To investigate the potential downstream effectors of miR-29a, we performed transcriptomic and proteomic profiling from 5XFAD and WT mouse hippocampi.

**Figure 5:**
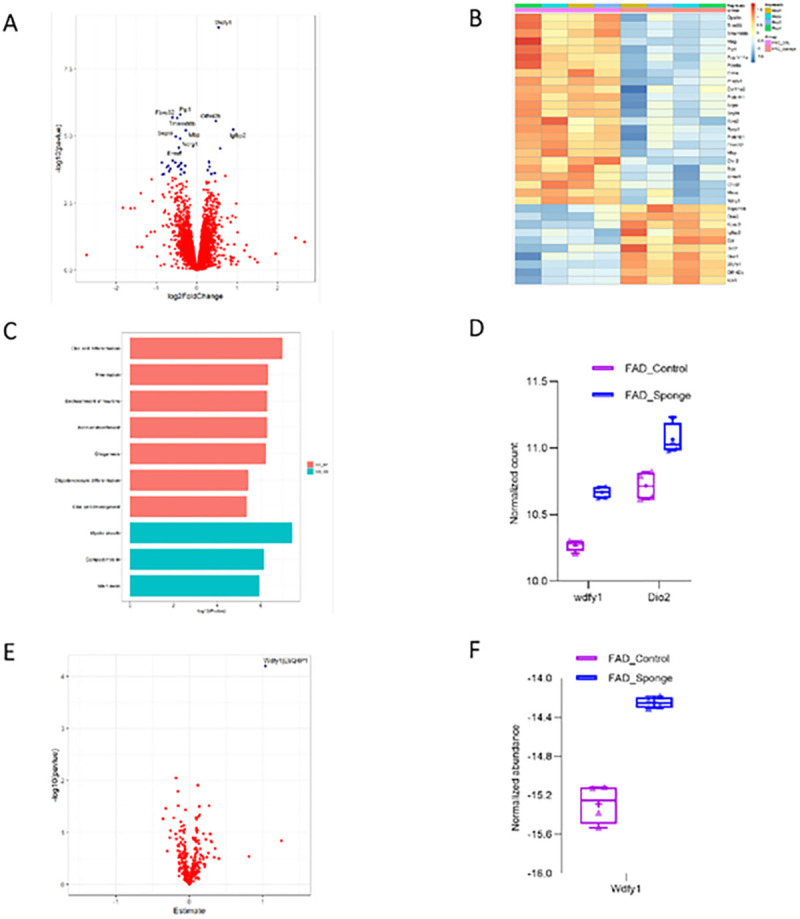
Downstream effectors of miR-29a at the transcript and protein level in 5XFAD mice. **A)** Volcano plot displaying the distribution of differentially expressed genes between FAD-Sponge and FAD-Control in the RNA-Seq analysis. Top10 DEGs were labeled. **B)**Heatmap of DEGs between FAD-Sponge and FAD-Control. Downregulated DEGs in FAD-Sponge vs. FAD-Control were depicted in blue and upregulated DEGs were in orange. **C)** GO enrichment analysis of downregulated DEGs in FAD-Sponge. **D)**Expression of DEGs that are predicted targets of miR-29a. **E)** Volcano plot displaying the distribution of differentially expressed proteins between FAD-Sponge and FAD-Control. **F)** Expression of the differentially expressed protein between FAD-Sponge and FAD-Control.

From transcriptomic profiling of 8 5XFAD and control mice, we found 34 transcripts that differed between control and miR-29a-sponge condition at FDR < 0.1. Of the 34 DEGs, 24 were down-regulated and 10 were up-regulated in FAD-Sponge compared with FAD-Control (Fig. 5A–B, Supplementary Table 2). To glean biological functions of the DEGs, we performed gene ontology (GO) enrichment analysis for down-regulated genes in FAD-Sponge that showed genes enriched in glial cell differentiation, myelination, ensheathment of neurons, and axon ensheathment at adjusted *p* value < 0.05 (Fig. 5C). Since miR-29a was down-regulated in FAD-Sponge, its downstream targets are expected to be upregulated. We found Wdfy1 and Dio2 were DEGs upregulated in FAD-Sponge and predicted to be targets of miR-29a^20^ (Fig. 5D), supporting them as putative targets of miR-29a.

**Figure 6:**
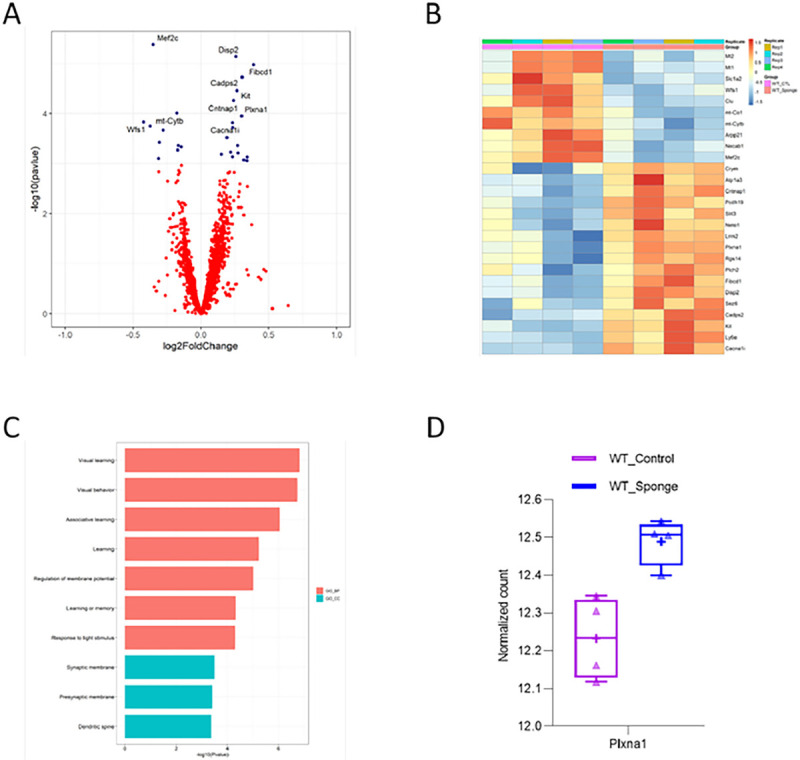
Downstream effectors of miR-29a at the transcript level in WT mice. **A)** Volcano plot displaying the distribution of differentially expressed genes between WT-Sponge and WT-Control. Top10 DEGs were labeled. **B)**Heatmap of DEGs between WT-Sponge and WT-Control. Downregulated DEGs in WT-Sponge vs. WT-Control were depicted in blue and upregulated DEGs were in orange. **C)** GO enrichment analysis of upregulated DEGs in WT-Sponge. **D)**Expression of DEG that is the predicted target of miR-29a.

From transcriptomic profiling of WT mice, we found 27 transcripts that differed between control and miR-29a-sponge status at FDR < 0.1. Of the 27 DEGs, 17 were up-regulated and 10 were down-regulated (Fig. 6A, Fig. 6B, Supplementary Table 3). GO analysis showed the up-regulated genes in WT-Sponge were enriched for genes implicated in visual learning, visual behavior, and associative learning (Fig. 6C). Similarly, the up-regulated genes in WT-Sponge were predicted miR-29a targets^20^. Plxna1 was found to meet both criteria (Fig. 6D) in WT mice.

We also sought to identify the downstream effectors of miR-29a at protein level since miRNAs alter gene expression either through mRNA degradation or translation inhibition. Thus, we performed proteomics on hippocampi using the same association testing as was used for the transcriptomic analyses. We imposed a completeness threshold of 50% proteins analyzed to limit the influence of less abundant proteins that are more likely to be incompletely sequenced in different samples. A total of 6559 proteins were used for differential expression analysis testing but we found no significantly differential proteins for FAD-Sponge vs. FAD-Control or WT-Sponge vs. WT-Control FDR < 0.01 (Supplementary Table 4–5). Since our goal is to identify the downstream targets of miR-29a in 5XFAD and WT mice, we next selected the proteins that are predicted targets based on miRDB database^20^. A total of 335 proteins were selected and used for further differential expression analysis. We found Wdfy1 was significantly up-regulated in FAD-Sponge compared with FAD-Control at FDR < 0.05 (Fig. 5E, Fig. 5F, Supplementary Table 6–7). Wdfy1 was also found to be up-regulated in FAD-Sponge at the transcript level, suggesting miR-29a could influence learning and memory in 5XFAD mice through WDFY1.

## Discussion

Our prior work identified miR-29a as strongly associated with cognitive decline in humans. To establish causal relationships, we investigated whether the loss of miR-29a function in mouse brain would influence memory, hippocampal pathology, and gene expression by delivering an AAV expressing miR-29a sponge to the hippocampus of 5XFAD and WT mice. Notably, administration of the miR-29a sponge to either WT or FAD mice improved some measures of learning and memory. The molecular consequences of lowering miR-29a levels were tested by measuring beta-amyloid deposition in FAD mice, and by measuring activated astrocytes and microglia by immunofluorescence staining in WT and FAD. We found beta-amyloid deposition was reduced in FAD mice receiving the miR-29a sponge, and measures of activated astrocytes and microglia were lower for WT and FAD mice receiving miR-29a sponge compared to their respective control groups. To identify potential molecular effectors of miR-29a, we profiled gene expression using transcriptomic and proteomic sequencing that identified Plxna1 and Wdfy1 as putative effectors at the transcript and protein level in WT and 5XFAD mice, respectively.

Previous studies showed that miR-29a increases with age across different species and tissues^27–31^, and miR-29a regulates age-dependent processes such as neuronal maturation and iron accumulation in the brain^32,33^. Increased miR-29a may be a normal response to escalating neuroinflammatory load^30^. In line with this notion, our data confirmed that endogenous expression of miR-29a increased in with age in WT mice. Interestingly, 2-month-old 5XFAD mice displayed miR-29a levels comparable to that of 12-month-old WT mice, and miR-29a expression remained essentially unchanged over time in the transgenics, raising the possibility of a dysfunctional response to increased brain inflammation in aged 5XFAD mice. We have previously reported that higher miR-29a levels were associated with faster cognitive decline in older people^5^. Consistent with prior findings, our data showed that miR-29a loss-of-function could improve some measures of learning and memory in both 5XFAD and WT mice. Immunofluorescence staining further indicated that miR-29a sponge attenuated the amyloid deposition and decreased activated astrocytes and microglia in the mouse hippocampus, highlighting the immunomodulatory role of miR-29a.

Transcriptomic and proteomic profiling designed to identify putative causal genes or sets of genes influenced by reduced miR-29a expression revealed different results in the WT and 5XFAD mice, although both showed alteration in genes targeted by miR-29a.

In 5XFAD mice, miR-29a sponge was significantly associated with genes involved in glial cell differentiation and myelination. Among them, PLP1, ERMN, MBP, and MAG are highly expressed in oligodendrocytes and are canonical oligodendrocyte markers^34^. In a recent study, the 5XFAD mouse transcriptomic signatures matched an inflammation-driven clinical AD subtype with an increase of oligodendrocyte and astrocyte markers^35^. We have also previously reported that decreased abundance of myelination-related proteins such as MBP was associated with cognitive stability in a proteome-wide association study^36^. Among miR-29a target genes in 5XFAD mice, WDFY1 and DIO2 were notably upregulated by expressing miR-29a sponge. Wdfy1 is a phosphatidylinositol 3-phosphate binding protein. Kong et al. reported that Wdfy1 attenuated neuroinflammation^37^, and they also found that forsythoside B attenuated memory impairment and neuroinflammation through increased Wdfy1 expression in an AD animal model. WDFY1 and WDFY family were also reported to be involved in neurogenesis, cerebral expansion and functional organization^38,39^. In the present study, WDFY1 was found to be up-regulated in miR-29 sponge-treated 5XFAD mice at both the transcript and protein level, suggesting it is a likely downstream target of miR-29a. Dio2 influences thyroid hormone action by converting the prohormone thyroxine (T4) to bioactive 3, 3’, 5-triiodothyronine (T3)^40^. Dio2 was found to be significantly down-regulated in AD^41^.

By contrast, miR-29a sponge expression in WT mice was associated with changes in genes involved in in visual learning, visual behavior, and associative learning. Among predicted targets of miR-29a, Plxna1 was differentially expressed. PLXNA1 is a transmembrane receptor mediating semaphorin signaling^42,43^ and has been reported to regulate the development of neurons and axonogenesis during early development^44,45^. Plexin signaling is believed to be involved in AD^45^. In our prior work, we also found that higher abundance of PLXNA1 is associated with cognitive stability in a proteome-wide association study of cognitive trajectory^5^.

We present additional evidence from mouse models of AD that miR-29a can modulate cognitive trajectory that we originally observed in human brains. The effects we observed on behavior were modest and most evident in 5XFAD mice. While we exclusively targeted our sponge to the hippocampus for miR-29a loss-of-function, it is possible that it contributes to cognitive function in other brain regions and that more widespread knockdown would have larger benefit. Interestingly, we observed that lowering miR-29a reduced beta-amyloid deposition in 5XFAD mice and measures of astrocyte and microglial activation in 5XFAD and WT mice. Transcriptomic and proteomic investigation of mouse brains points to putative targets of miR-29a. These targets differ in 5XFAD and WT mice, which cannot be explained by these data. Further study of WDFY1 and PLXNA1 in different cell types would likely shed light onto the context miR-29a acts to ameliorate cognitive decline with age.

## Data Availability

Upon publication phenotypic, transcriptomic, and proteomic data used in this manuscript will be available at Synapse ID: syn51236902. Supplementary Tables are available on Synapse at syn51369737.
